# Polyamine impact on physiology of early stages of reef-building corals–insights from rearing experiments and RNA-Seq analysis

**DOI:** 10.1038/s41598-024-72943-6

**Published:** 2024-10-08

**Authors:** Kodai Gibu, Nanami Mizusawa, Mariko Iijima, Yoshikazu Ohno, Jun Yasumoto, Ko Yasumoto, Akira Iguchi

**Affiliations:** 1 Geological Survey of Japan, National Institute of Advanced Industrial Science and Technology (AIST), 1-1-1 Higashi, Tsukuba, Ibaraki 305-8567 Japan; 2 Department of Ecosystem Studies, Graduate School of Agricultural and Life Sciences, The University of Tokyo, Yayoi, 113-0032 Japan; 3 School of Marine Biosciences, Kitasato University, 1-15-1 Kitasato, Minami, Sagamihara, Kanagawa 252-0373 Japan; 4 Faculty of Agriculture, University of the Ryukyus, 1 Senbaru, Nishihara, Nakagusuku, Okinawa 903-0213 Japan; 5 Research Laboratory On Environmentally-Conscious Developments and Technologies [E-Code], National Institute of Advanced Industrial Science and Technology, Tsukuba, Ibaraki 305-8567 Japan

**Keywords:** Ecophysiology, Environmental impact

## Abstract

Polyamines are involved in various functions related to the cellular-level responses. To assess effects of polyamines on marine organisms, rearing experiments and comprehensive gene expression analyses were conducted on *Acropora digitifera* and *Acropora* sp.1, representative reef-building corals along the west-central coast of Okinawa, Japan, to evaluate effects of putrescine. Concentrations of putrescine ≥ 1 mM dissolved tissues of juvenile polyps and increased mortality of planula larvae. RNA-Seq analysis of juvenile polyps exposed to putrescine at the stage before effects became visible revealed dynamic fluctuations in gene expression in the putrescine-treated samples, with increased expression of stress-responsive genes (e.g. NAD-dependent protein deacylase sirtuin-6) and the polyamine transporter Slc18b1-like protein. These results also suggest that putrescine affects expression of genes related to ribosomes and translation. This study provides important insights into roles of polyamines and future directions regarding physiological responses of corals.

## Introduction

Polyamines are aliphatic compounds having more than two amino groups. They participate in various essential cellular functions in human cells, including cell proliferation and intracellular signaling^1^. Polyamines have been investigated in relation to plant morphogenesis and stress responses^2–4^, and are recognized as essential for bacterial growth and biofilm formation^5,6^, as well as for proliferation in archaeans^7^. While polyamines serve physiologically important functions, high concentrations of exogenous polyamines exhibit cytotoxicity in experiments with yeast and human cells^8,9^. Polyamines also capture CO_2_ and contribute to formation of calcium carbonate^10,11^, suggesting that polyamines are involved in essential coral processes, but the small number of taxa investigated limits the extent to which findings can be generalized to other groups.

Reef-building corals are keystone organisms of biodiverse coral reef ecosystems, and support biotech, fisheries, and tourism industries^12^. Also, coral reefs provide shelter for many organisms, and protects seashores from erosion^13^. Although corals and other cnidarians are among the simplest extant animals, they share many genes with vertebrates^14,15^, making it easy to apply functional genetic information from vertebrates.

Corals also produce calcium carbonate skeletons, forming the structural basis of coral reefs. Corals accumulate various ions from seawater to create aragonite skeletons in calcifying tissue^16^, where greatly enhanced concentrations of calcium and carbonate ions contribute to calcification. Using a cell-impermeable fluorescent dye as a tracer, it can be seen that seawater flows into the calcifying space via the paracellular pathway during skeleton formation^17^^,^^18^^,^^19^. As previously mentioned, a correlation between polyamines and coral calcification has been proposed. Introduction of polyamines into seawater via coral polyps permits assessment of the impact of polyamines on coral calcification sites. Since recruitment, i.e., settlement of planula larvae, is essential for maintenance of coral populations, as the first step in the life history of corals, it is important to evaluate motile planula larvae and sessile coral polyps in terms of environmental impacts^20^.

In this study, we aimed to evaluate effects of polyamines on physiological aspects of marine organisms using corals of the genus *Acropora*, a representative group of reef-building corals in rearing experiments and gene expression analysis. To assess physiological effects of polyamines on corals, planula larvae and juvenile polyps of *Acropora digitifera*^21^, which is dominant in the Ryukyu Islands, and *Acropora* sp.1, a cryptic species of *A. digitifera*^22^, were exposed to polyamines and subjected to comprehensive gene expression analysis using RNA-Seq. The morphology and behaviour of planula larvae and juvenile polyps of these *Acropora* species are very similar. As physiological mechanisms involving polyamine are unknown, experiments were conducted using putrescine. Through a series of experiments, we assessed physiological consequences of coral exposure to putrescine to better understand polyamine metabolic functions related to coral physiology (energy, DNA translation, detox, etc.).

## Results

### Impact assessment study

**Fig. 1:**
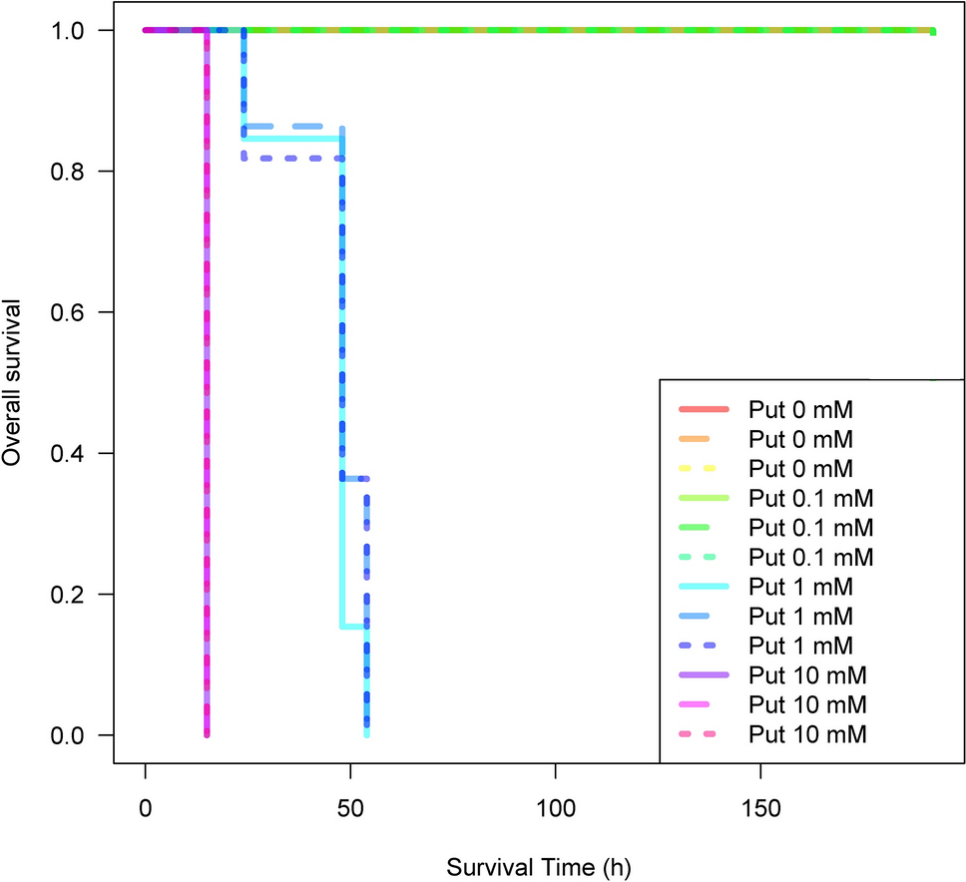
Survival of planula larvae of *Acropora digitifera* reared on 0 mM, 0.1 mM, 1 mM or 10 mM putrescine for 7 days at 25 °C. Approximately 20 planula larvae were tested and three sets at each concentration were set up, 12 test plots in total.

**Fig. 2:**
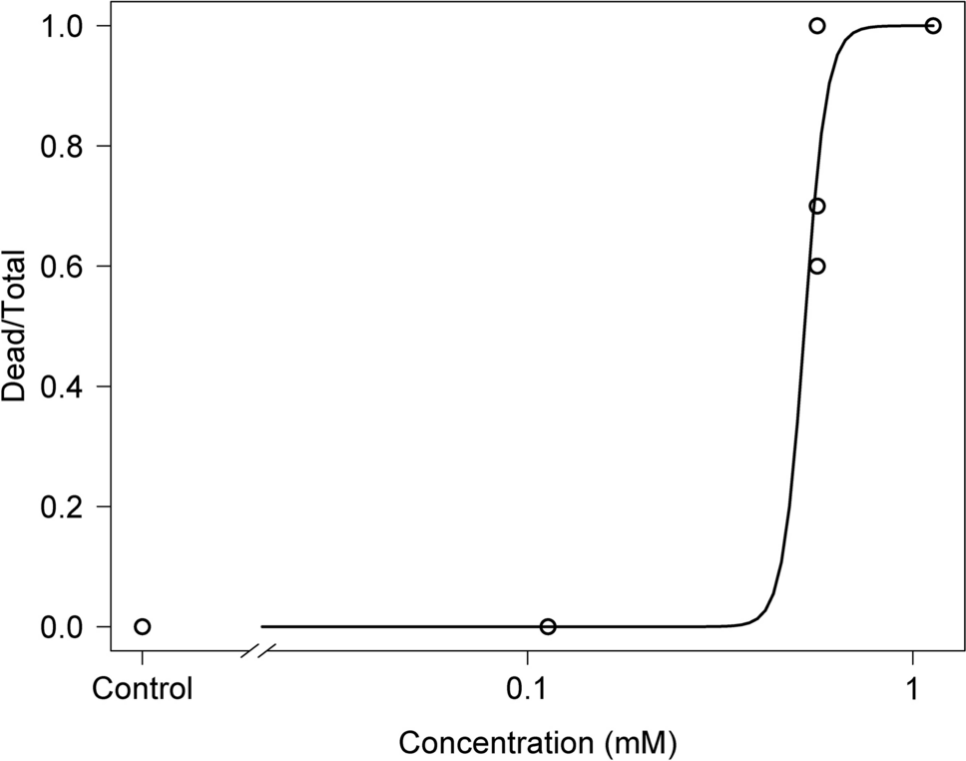
Response curve to putrescine in planula larvae of *Acropora digitifera* in a rearing experiment for 72 h, with putrescine concentrations of 0 mM, 0.113 mM, 0.565 mM or 1.13 mM.

**Fig. 3:**
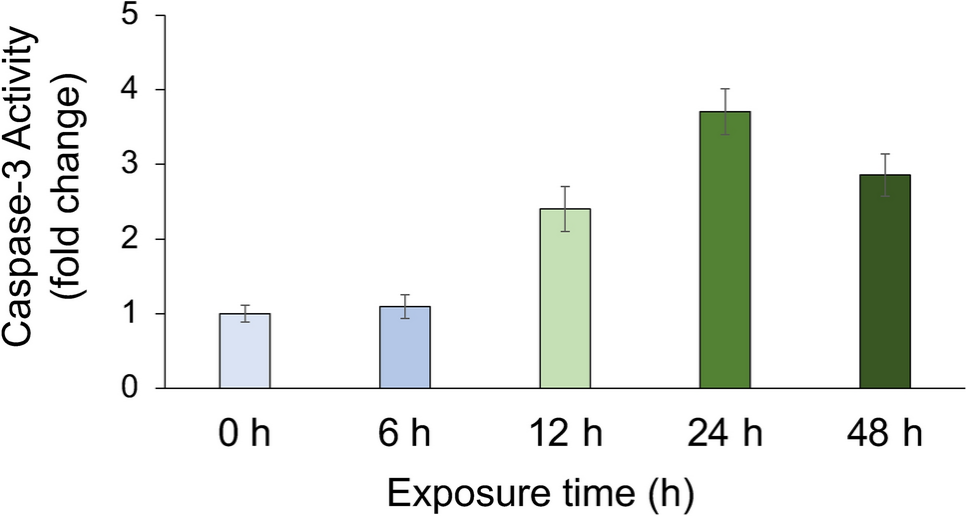
Caspase-3 activity measurements in juvenile polyps of *Acropora digitifera* reared in seawater with 10 mM putrescine, every 0, 6, 12, 24 and 48 h.

As a first step in assessing effects of polyamines on corals, we examined survival of planula larvae following exposure to putrescine. No planula larvae treated with 0.1 mM putrescine died (Fig. 1), but those treated with 1 mM putrescine all died within 63 h after addition, and at 10 mM putrescine all individuals were dead after 15 h (Fig. 1) (p < 0.01, log-rank test). The LC_50_ of putrescine in planula larvae in a 72-h rearing experiment was 0.524 mM (Fig. 2). As a result of morphological observations on sessile juvenile coral polyps exposed to putrescine, polyps treated with 10 mM putrescine began to show loss of tissues at 12 h, and all individuals showed dissolved tissues by 48 h (Fig. S1). In a Caspase-3 activity test of juvenile polyps treated with 10 mM putrescine, activity increased 12 h after addition and was maximal after 24 h (Fig. 3).

### Expression analysis by RNA-Seq

**Fig. 4:**
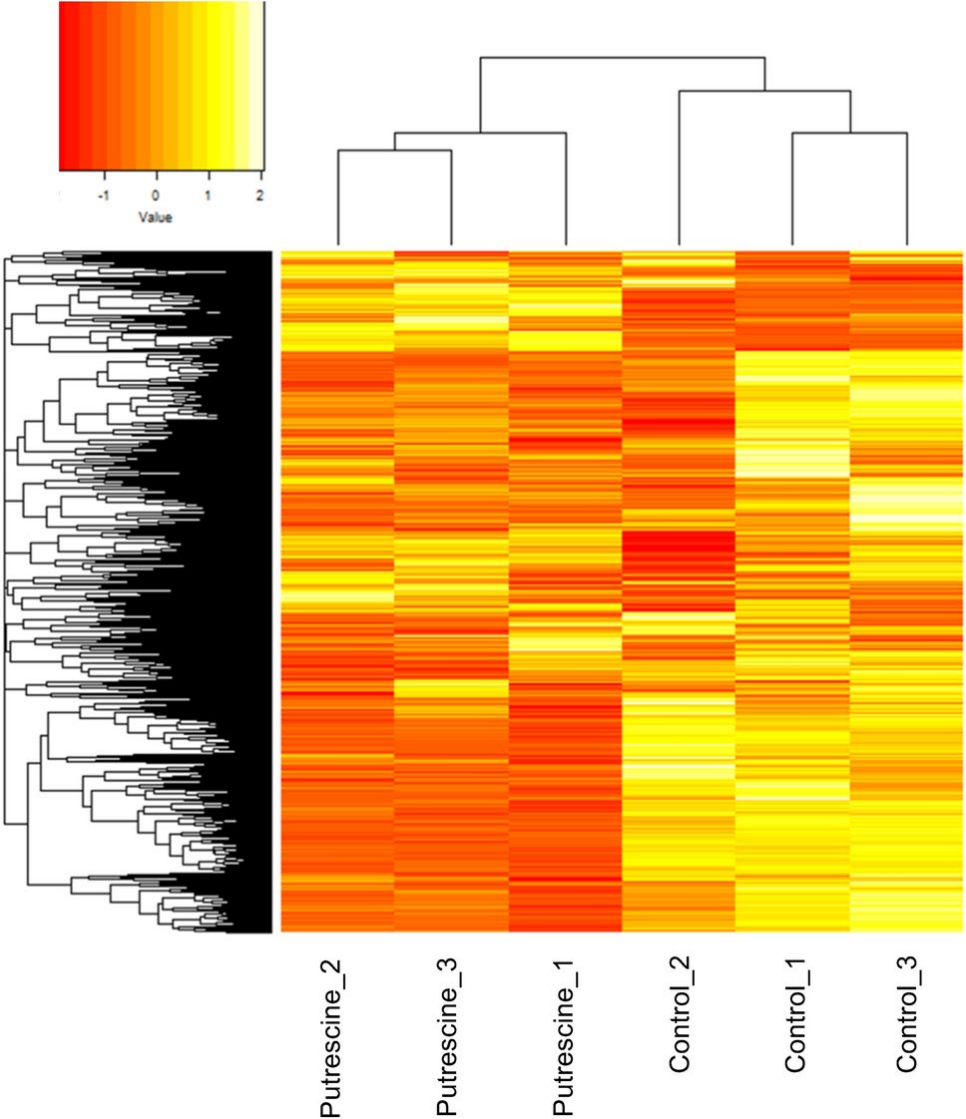
Heatmap of gene expression pattern of *Acropora* sp.1; X-axis: total 6 samples (3 control and 3 10 mM putrescine-treated (6 h exposure) samples; Y-axis: expression pattern of all genes.

**Fig. 5:**
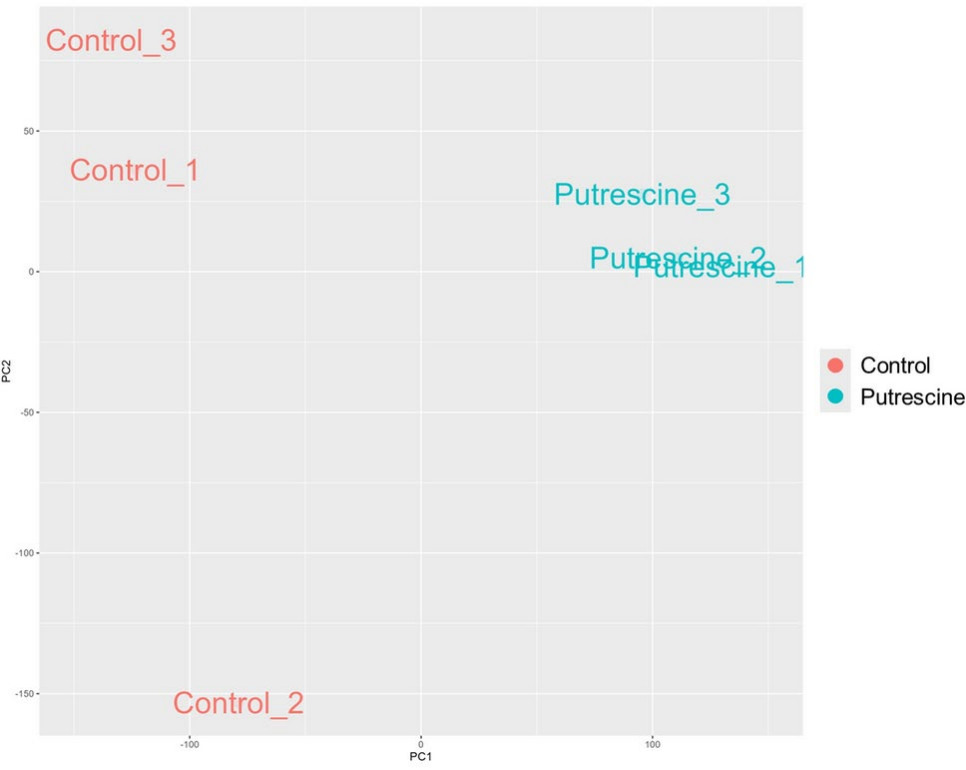
PCA plots of gene expression pattern of *Acropora* sp.1 for three control samples and three 10 mM putrescine-treated samples.

In total, 30,819 transcripts were constructed, of which 21,569 were annotated in the SwissProt database (Table S2). Expression analysis identified 3,924 transcripts as differentially expressed genes (DEGs) (Table S3), of which 2,369 genes showed higher expression after putrescine treatment and 1,555 genes showed lower expression compared to controls (Table S3). A heatmap showed different gene expression patterns between controls and putrescine treatments (Fig. 4). Cluster and principal component analyses formed two large clusters, putrescine-treated and controls (Figs. 5 and S3). GO enrichment analysis did not detect any significantly different GO terms between control and putrescine-treated samples.

## Discussion

Previous studies have reported that excessive accumulation of polyamines, including putrescine, can be toxic to organisms^9,23^, and this also applies to corals, as our tests assessing effects of polyamines on *Acropora* sp. 1 planula larvae and juvenile polyps show (Figs. 1, 2, and S1). In juvenile polyps, exposure to putrescine increased activity of Caspase-3, an important enzyme in the apoptotic pathway (Fig. 3). Excessive accumulation of putrescine has been reported to induce apoptosis^24,25^, and our results confirm those findings.

Transcriptome analysis provides a comprehensive assessment of gene expression and allows environmental responses of organisms to be examined from a molecular perspective. Juvenile polyps treated with putrescine showed significant changes in gene expression (Figs. 4 and 5). Gene expression in the control treatment varied between samples, probably due to the influence of micro-organisms, but for the putrescine treatment, variation between samples was small, giving a glimpse of the magnitude of the effect. Expression of polyamine transporter Slc18b1-like protein also increased under polyamine treatment (Table S3, Asp1_24168). Slc18b1is reportedly involved in regulation of intracellular polyamine levels^26^ and may contribute to the coral response to putrescine observed in this study. Genes such as plasma-membrane calcium-transporting ATPase (PMCA), calcium channel protein, and calmodulin were among the DEGs (Tables S4—S6), and these genes are important for calcium metabolism and calcification of reef-building corals^27–29^. PMCA was up-regulated by putrescine treatment (Table S4), whereas calcium channel protein and calmodulin were up- and down-regulated, respectively (Tables S5 and S6). Eukaryotic translation initiation factor 2-alpha kinase 3, implicated in the skeletal growth of corals^30^, was also identified among the DEGs list (Table S3, Asp1_29210). These findings suggest that putrescine may have a complex effect on calcification, but detailed studies will be required to determine the mechanism of action by measuring calcification rates of corals treated with putrescine and by assessing gene expression using quantitative PCR.

A number of genes with GO numbers for translation or ribosomes were found among DEGs including GO:0,003,735: Asp1_07129, Asp1_05683; GO:0,006,412: Asp1_07520, Asp1_08567). The transcriptome included in these GO terms primarily codes for genes such as ribosomal subunits (Asp1_07129, Asp1_05683, Asp1_08567) and tRNA synthetase (Asp1_07520), which are known to function in the translation process^31^. Experiments with cyanobacteria have shown that putrescine adsorbs to ribosomal subunits and stops protein synthesis^32^. In corals, putrescine has the same effect. Several DEGs with NAD-dependent protein deacetylase activity (GO: 0,034,979) were also found (Asp1_05519). NAD-dependent protein deacetylase activity is important in cell survival and stress responses^33^ and is activated under stress conditions in marine organisms^34^. For reef-building corals, exposure to polyamines may also trigger a stress response, but further physiological investigations using captive specimens and cultured cell system^35^ are needed to identify detailed mechanisms of putrescine. For morphological observations, it will be necessary to carry out histological observations using fluorescence microscopy on polyamine-exposed samples and to assess the metamorphosis and settlement ability of larvae. Evaluation of expression of Slc18b1-like proteins found in this study by quantitative PCR is expected to facilitate future assessments of polyamine effects on corals.

## Materials and methods

### Tests to evaluate effects of polyamines using *Acropora* species

Planula larvae and juvenile polyps of *Acropora digitifera* and *Acropora* sp.1 were used in polyamine addition experiments. The polyamine chosen was putrescine (MW 88.15 g/mol) (1,4-butanediamine, Fujifilm-Wako, Osaka, Japan), the major biological diamine. Putrescine is a precursor for triamines and tetraamines, such as spermidine (MW 145 g/mol) and spermine (MW 202 g/mol). *Acropora digitifera* colonies were collected at the reef edge near Sesoko Island, Okinawa, Japan. *Acropora* sp. 1 colonies were collected at the reef edge of Bise, Okinawa, Japan. Coral colonies were collected with permission from Okinawa Prefecture (permit numbers: 3–72, 4–11 and 4–20). For *A. digitifera*, 10 spawning colonies were used for collecting gametes in June 2022. For *Acropora* sp.1, as with *A. digitifera*, gametes were collected from six spawning colonies in August 2022. Planula larvae were secured by fertilization of collected gametes. Basic protocols for preparation of juvenile polyps from planula larvae were carried out according to our previous study^36,37^. Juvenile polyps were obtained by exposing planula larvae (21–63 days old) to 10 µM metamorphic peptide (Hym-248^38^; Eurofins Genomics KK, Tokyo, Japan) for 42 h and allowing them to metamorphose and settle.

Approximately 20 planula larvae (56 days old) of *A. digitifera* were placed in 3 mL of filtered seawater, and test sections were prepared with putrescine at concentrations of 0 mM, 0.1 mM, 1 mM and 10 mM. Three replicates were conducted for each test. These were reared at 25 °C for 7 days and surviving individuals were counted to determine survival rates. A Kaplan–Meier curve for putrescine in planula larvae was calculated and significant differences were confirmed by the log-rank test using R version 4.2.2^39^, packages *survival*, version 3.5.7^40^. In addition, survival rates were calculated for three replicates of approximately 20 planula larvae each, as above, with rearing experiment for 72 h based on putrescine concentrations of 0 mM, 0.113 mM, 0.565 mM and 1.13 mM, and Lethal Concentration 50 (LC_50_) was determined using R package *drc*, version 3.0.1^41^. The concentration range was set with reference to a preliminary study using 0.001 mM–0.1 mM putrescine.

Approximately 20 coral juvenile polyps (63 days old, 24 days after settlement) of *A. digitifera* were added to wells containing seawater and putrescine concentrations of 0, 0.1 mM and 10 mM, and three replicates of each test were prepared. These were kept for 48 h and morphological observations were made under a M165FC stereomicroscope (Leica, Wetzlar, Germany) at 0, 6, 12, 24 and 48 h. In parallel, a Caspase-3 assay was performed using an APOPCYTO Caspase-3 Colourimetric Assay Kit (MBL, Japan) and its protocol 12 h after addition of 10 mM putrescine to 20 juvenile coral polyps. Caspase-3 activity was calculated by determining the fold change from the control.

### RNA extraction and sequencing

Approximately 100 juvenile polyps (21 days old, 2 days after settlement) of *Acropora* sp.1 were reared in five 10-mM putrescine-treated samples with five controls at room temperature for 6 h. The 10 mM, 6-h putrescine treatment was prepared based on morphological observations and Caspase-3 activity results, which indicated that these samples were just before effects of putrescine began to appear. After rearing, total RNA was extracted from juvenile polyps using ISOGEN (Nippon Gene, Tokyo, Japan) and extraction was performed according to the ISOGEN manual. RIN values were measured with a BioAnalyzer (Agilent Technologies, Inc.) and three RNA samples with high RIN values were selected for the control and putrescine-treated samples, respectively (three replicates for each test). Libraries were prepared using an MGIEasy RNA Directional Library Prep Set (MGI Tech Co., Ltd., Shenzhen, China), and were sequenced using an MGI DNBSEQ-G400RS (MGI Tech Co. Ltd.; 100-bp paired-end reads) for sequencing.

### Bioinformatics

Trimmomatic version 0.39^42^ was run with default values and trimming was performed for obtained sequences. Resulting reads were assembled using Trinity, version 2.13.2^43^ and assembled DNA sequences were translated into amino acid sequences. Redundant amino acid sequences were removed with CD-HIT, version 4.8.1^44^ (-c 0.95). Predicted amino acid sequences were filtered as coral-derived sequences by running Blastp version 2.14.1^45^ (e-value < 1e^-5^) against *Acropora digitifera* protein sequences^46^. The transcriptome was filtered for coral-derived sequences. The transcriptome was annotated by running Blastp (e-value < e^-5^) on the SwissProt protein database (UniProt release 2023_04). Trimmed reads were aligned to the transcriptome sequence using Kallisto, version 0.50.0^47^.

### Data analysis

Heat maps were created using scaled TPM values (mean 0, standard deviation 1) with the gplots package^48^ in R version 4.2.2^39^ based on TPM values output from Kallisto. Cluster analysis was also performed based on Ward.D2 with 1,000 bootstraps using the pvclust package^49^ in R. Subsequently, non-hierarchical cluster analysis was performed using the K-medoids method in the package cluster, version 2.1.4^50^ to calculate silhouette values. The number of clusters was estimated from calculated silhouette values and principal component analysis was performed to examine dissimilarities between samples.

Transcript level abundance and counts were loaded in tximport version 1.24.0^51^ and summarized to the gene level. Samples with normalized counts < 10 were removed. Differentially expressed genes (DEGs) were compared between three test and three control samples treated with 10 mM putrescine using DESeq2 version 1.36.0^52^. DESeq2 was run with default parameters, p < 0.05 Gene ontology enrichment analysis was performed using GOSeq version 1.48.0^53^. Each GO term was tested and a threshold (p < 0.05) was used to identify GO terms that were differentially expressed. Tests were corrected for gene length to account for bias due to differences in gene length. Over-represented p-values were also applied to the FDR value obtained with Benjamini–Hochberg correction to identify GO terms that were significantly variable with a cut-off of FDR < 0.05.

## Supplementary Information

Supplementary Information 1.

Supplementary Information 2.

Supplementary Information 3.

## Data Availability

Sequence data obtained (details in Table S1) were registered with the DNA Data Bank of Japan (accession: DRA017512).
