# Supplementary material for: Polyamine impact on physiology of early stages of reef-building corals–insights from rearing experiments and RNA-Seq analysis
Source: Sci Rep. 2024 Oct 8;14:23465. doi: 10.1038/s41598-024-72943-6 (PMC11461621; doi:10.1038/s41598-024-72943-6)
Supplement: Supplementary file 1 — Supplementary Information 1. [file 41598_2024_72943_MOESM1_ESM.pdf]

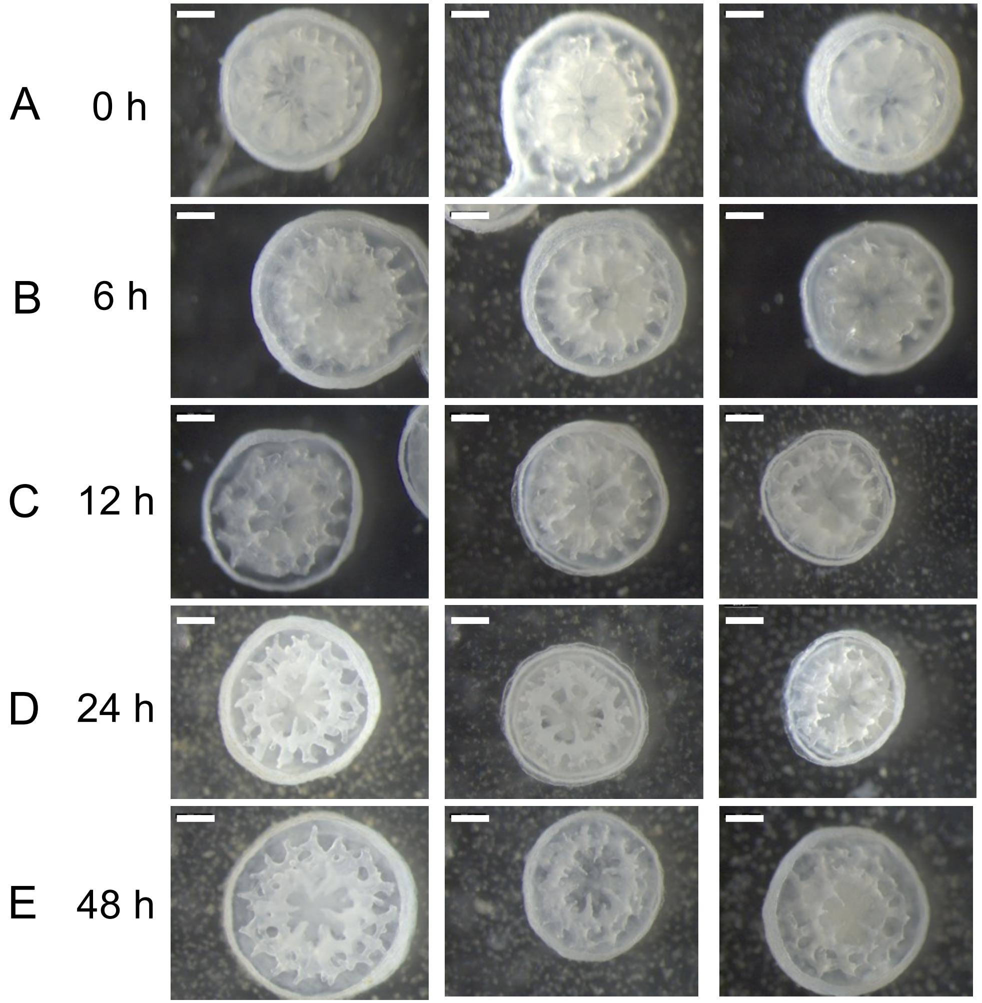


Figure S1. Morphological observations of juvenile polyps of *Acropora digitifera* reared with 10 mM putrescine and performed every 0, 6, 12, 24 and 48 h. Three individuals are shown at each time. A, Tissues and mouths were clearly observed. B, No change was observed at 6 h. C, Skeletons began to be noticeable at 12 h. D, Skeletons were noticeable and tissues were dissolving. E, All tissues were completely dissolved. G, All tissues were completely dissolved. Animals were considered dead at this point. Scale bars indicate 200 µm.
