# Supplementary material for: Polyamine impact on physiology of early stages of reef-building corals–insights from rearing experiments and RNA-Seq analysis
Source: Sci Rep. 2024 Oct 8;14:23465. doi: 10.1038/s41598-024-72943-6 (PMC11461621; doi:10.1038/s41598-024-72943-6)
Supplement: Supplementary file 2 — Supplementary Information 2. [file 41598_2024_72943_MOESM2_ESM.pdf]

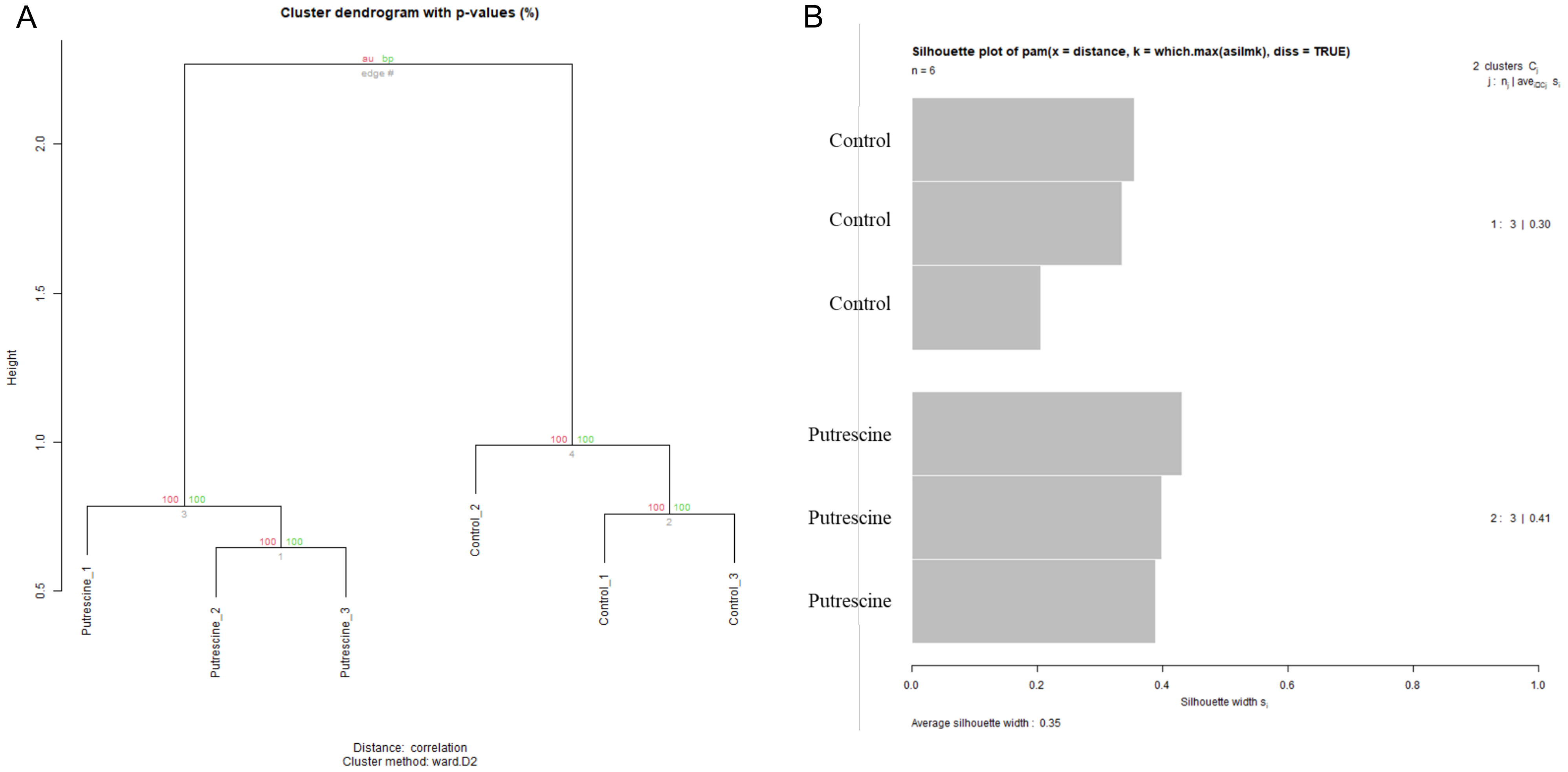

Figure S2. Results of cluster analysis using three control and three 10-mM putrescine-treated samples of *Acropora* sp.1. A: Cluster analysis by pvclust. Red letters indicate approximately unbiased p-values. Green letters indicate bootstrap probability values. B: Silhouette values from non-hierarchical cluster analysis.
